# A Permutation-Invariant, Missing-View-Aware Multi-Perspective Recurrent Neural Network Cell for Time-Series Fusion

**DOI:** 10.3390/s26196330

**Published:** 2026-10-07

**Authors:** Chao Han, Ruidong Wang, Lilong Zhang

**Affiliations:** School of Electrical and Information Engineering, North Minzu University, Yinchuan 750021, China; wangruidong3366@163.com (R.W.); zhanglilong1214@163.com (L.Z.)

**Keywords:** multi-perspective recurrent neural networks, multi-source time series, order invariance, missing-view robustness, activity recognition, sensor fusion

## Abstract

Recurrent models for multi-source data must combine synchronized streams while remaining usable when sources are reordered or unavailable. Multi-perspective long short-term memory (MP-LSTM) cells update a joint memory across perspectives, but a sequential update can make the result depend on source order. We implement a generalized chain comparator and replace its perspective-wise chain with shared projections and set attention over available views. All experiments use one fixed subject-independent split of the UCI Daily and Sports Activities (DSA) dataset, with subjects 7 and 8 as the two held-out test participants. On this split, the proposed cell uses 66.0% fewer parameters and has a 41.5% shorter recorded training time than the comparator. On a fixed 720-segment, seed-0 test subset, its inference-time accuracy remains 75.14% across eight random view permutations (standard deviation (SD) 0.00 percentage points), whereas the chain comparator falls from 76.39% to a mean of 24.67%. In the 10-class task, the mean full-view accuracy across three training seeds is 3.53 percentage points lower for the proposed cell. A cyclic missing-view schedule increases seed-0 accuracy with four of five views removed from 17.47% to 41.41% in the 19-class task, while three-seed mean full-view accuracy decreases by 3.40 percentage points. These results characterize order-invariant fusion and its robustness–accuracy trade-off in this controlled case study; they do not establish generalization beyond this dataset and held-out-subject split.

## 1. Introduction

Human activity, speech, and many physical processes are observed through multiple synchronized sources. A scene can be recorded by several cameras, movement by inertial units placed on different body segments, and speech by concurrent acoustic and visual signals. Because no single source provides a complete description, a useful representation must capture temporal structure within each source while combining complementary information across sources. Recent surveys discuss multimodal integration at the representation and decision levels [1,2,3]; sensor-level fusion also requires attention to alignment, calibration, uncertainty, and source failure [4,5,6]. Multi-view learning formulates related problems in terms of information shared across views and information that remains view-specific [7,8,9,10]. In wearable activity recognition, the synchronized multi-source input consists of accelerometer, gyroscope, and magnetometer channels attached at different body locations [11,12,13,14,15,16,17,18,19].

Gated recurrent networks are well suited to aligned time series. Long short-term memory (LSTM) introduced memory cells for learning long-range dependencies [20]; forget gates subsequently improved the treatment of continuous input streams [21], and bidirectional variants incorporate both past and future context [22]. In our comparator, each perspective computes input, forget, output, and candidate-memory gates from its own input and the shared hidden state. The perspective updates are then applied in a fixed chain:

(1) $$\begin{array}{cc} c_{1} & =f_{1}c_{t-1}+i_{1}{\tilde{c}}_{1},\\ c_{2} & =f_{2}c_{1}+i_{2}{\tilde{c}}_{2},\\ \; & \;\;\vdots \\ c_{V} & =f_{V}c_{V-1}+i_{V}{\tilde{c}}_{V},\\ c_{t} & =c_{V}. \end{array}$$

Each perspective reads the jointly updated memory through its output gate, and the cell output is formed by aggregating the resulting hidden states.

Multi-perspective recurrent fusion raises three practical issues. Many formulations assume a fixed number of views and feature dimensions, which complicates transfer across sensor configurations and input modalities. Sequential updates also make the learned representation sensitive to the chosen source index: reordering otherwise identical inputs can change the output. Finally, incomplete observations are often handled with ad hoc masking or source-specific imputation, leaving the recurrent model without a general rule for maintaining a joint representation when views are unavailable.

Public wearable-activity benchmarks make this setting reproducible across sensor placements and hardware [7,11,19,23,24]. The UCI DSA dataset provides synchronized inertial and magnetic measurements from five body-worn units [25], while related body-worn datasets offer independent evaluation protocols [5,19]. We implement a multi-perspective recurrent cell and extend it to handle source-order changes and missing views:

1. We generalize the cell to an arbitrary number of views and arbitrary input dimensions.
2. We replace per-perspective cell parameters with shared projections, which makes the gate computations permutation equivariant.
3. We merge per-view candidate updates with a set-attention operation whose query is the current joint memory.
4. We evaluate missing-view masks and leave-one-view ablations under a common subject-independent protocol on a public sensor dataset.
5. We add a cyclic missing-view schedule and missing-aware validation that recover part of the severe missing-view gap without reintroducing source-specific parameters.

This study evaluates permutation-invariant fusion and missing-view handling under a fixed subject-independent protocol on one real-world dataset. We report the observed benefits together with the associated performance trade-offs, so the evidence is tied to the evaluated implementations and this dataset.

The methodological distinction is an architectural constraint, not an additional attention block. The cell applies the same gate map to every available view, forms local memory candidates from the same previous state, and reduces those candidates with a mask-aware symmetric operator before updating the joint state. The recurrent update is therefore invariant to view indexing. We include shared projection followed by attention and a conventional temporal encoder as a separate baseline because input-level set pooling does not test invariance of the recurrent memory update. The contribution is a deterministic order-invariant recurrent fusion cell together with a direct measurement of its robustness–accuracy trade-off. On the evaluated split, full-view accuracy is lower than that of several baselines, and severe view dropout remains more difficult for the proposed cell than for the generalized chain comparator.

## 2. Related Work

Multi-source sequence recognition can be organized by when source information is combined [1,2,3]. Early fusion concatenates per-frame source representations before temporal modeling. Late fusion trains separate sequence encoders and combines their final representations or scores. Intermediate fusion exchanges information between sources during temporal processing. The same distinction appears in multi-view learning, where the key question is which information is shared across views and which remains view specific [7,8,9,10].

Deep recurrent, convolutional, and attention-based models are widely used for wearable activity recognition [5,6,7,11,12,13,14,15,16,17,18,19,23,24,26,27]. They commonly concatenate body-worn sensor channels, use one network per sensor, or place a convolutional front end before recurrent temporal modeling. These choices have improved accuracy on public benchmarks, but they generally assume a fixed sensor set with a meaningful order.

The chain comparator updates a joint cell state one perspective at a time. The proposed model retains this recurrent fusion setting while removing the assumption that source order is meaningful. In sensor and camera networks, a source may become unavailable, a camera may be physically repositioned, or source indices may be assigned arbitrarily. A fusion cell whose output changes under such relabeling is poorly matched to these deployment conditions.

Set-processing research has established the value of permutation-invariant decoders, random-permutation reasoning, and invariant neural representations [8,9,10]. Attention-based multi-view fusion provides a complementary way to select and weight source content dynamically [7,28]. Recent multimodal studies also address missing views through contrastive masked attention, modality translation, graph completion, and federated learning for incomplete modalities [29,30,31,32,33]. Robust recurrent models have been applied to degraded or faulty sensing; for example, a conditional LSTM autoencoder was proposed for fault diagnosis in nonlinear unmanned-aerial-vehicle systems [34]. Together, these studies motivate a design that combines shared recurrent projections, attention-based aggregation over available views, and an explicit availability mask.

We compare the proposed cell with the following baselines under a common protocol:

- Concat + bidirectional long short-term memory (BiLSTM), an early-fusion baseline;
- Per-view BiLSTM, a late-fusion baseline;
- Generalized chain comparator, a PyTorch 2.14.0+cu132 model inspired by the perspective-by-perspective memory update;
- Chain comparator trained with random view permutations, which simulates a data-augmentation attempt to tolerate order variability.
- Shared-set attention + BiLSTM, which applies shared projection and masked attention before a standard temporal recurrent encoder.
- Set Transformer + BiLSTM, a self-attention-based set encoder with the same temporal and classification dimensions.

The study provides a controlled assessment of how permutation invariance affects the performance and robustness of a multi-perspective recurrent fusion cell. We therefore examine the benefits and trade-offs of removing dependence on source ordering, with benchmark ranking as a secondary consideration.

## 3. Method

### 3.1. Input Representation

Let a multi-source sequence be represented by

(2) $$x=\left(x_{1},\ldots ,x_{T}\right),\;x_{t}\in R^{V\times F},$$

Here *T* denotes the number of time steps, *V* the number of perspectives, and *F* the feature dimension of each perspective. A missing source is represented by a Boolean mask $m_{t}\in {\left\{0,1\right\}}^{V}$, where $m_{t,v}=1$ indicates that perspective *v* is unavailable. Missing values are zero-filled before they enter any model, and availability indicators are provided to the two BiLSTM baselines.

### 3.2. Generalized Chain Comparator

The chain comparator retains the perspective-wise memory update and LSTM gate structure [20,21]. It is a generalized comparator, not an implementation-equivalent reproduction of a released three-view model. It uses a separate recurrent projection for each view, supports arbitrary view counts and masks, and averages the outputs of present views rather than summing them. The comparisons below therefore concern the implementations evaluated here. For perspective *p*, the comparator computes:

(3) $$\begin{array}{cc} i_{p} & =\sigma \left(W_{i}^{p}x_{p}+U_{i}^{p}h+b_{i}^{p}\right), \end{array}$$

(4) $$\begin{array}{cc} f_{p} & =\sigma \left(W_{f}^{p}x_{p}+U_{f}^{p}h+b_{f}^{p}\right), \end{array}$$

(5) $$\begin{array}{cc} {\tilde{c}}_{p} & =\tanh\left(W_{c}^{p}x_{p}+U_{c}^{p}h+b_{c}^{p}\right), \end{array}$$

(6) $$\begin{array}{cc} o_{p} & =\sigma \left(W_{o}^{p}x_{p}+U_{o}^{p}h+b_{o}^{p}\right). \end{array}$$

The perspective states are then applied in source order. After this loop, each perspective reads the final memory $c_{t}$ through its output gate:

(7) $$h_{p}=o_{p}\tanh\left(c_{t}\right),$$

The cell returns the mean of the $h_{p}$ values from the present perspectives.

### 3.3. Robust MP-LSTM Cell

The proposed cell retains the idea that each perspective proposes a memory update and reads a shared memory, while removing two order-dependent components.

First, all perspectives share the input projection, hidden-state projection, bias, and peephole parameters. With separate parameters, the computation would depend on which physical source is assigned to each parameter index after a permutation. Shared projections instead make the gate computation equivariant to source reordering.

Second, the cell does not apply perspective updates sequentially. For each present perspective *p*, it computes the shared gate outputs $i_{p}$, $f_{p}$, ${\tilde{c}}_{p}$, and $o_{p}$, and then forms a local candidate update:

(8) $$c_{p}^{\mathrm{local}}=f_{p}c_{t-1}+i_{p}{\tilde{c}}_{p}.$$

The set of local candidates is then passed through three shared linear projections:

(9) $$\begin{array}{cc} q & =Q(c_{t-1}), \end{array}$$

(10) $$\begin{array}{cc} k_{p} & =K(c_{p}^{\mathrm{local}}), \end{array}$$

(11) $$\begin{array}{cc} v_{p} & =V(c_{p}^{\mathrm{local}}). \end{array}$$

Attention weights are computed only over present perspectives, using the scaled dot-product form adopted in recent attention-based fusion models [7]:

(12) $$\alpha_{p}={\mathrm{softmax}}_{p}\;\left(\frac{q^{⊤}k_{p}}{\sqrt{H}}\right),$$

The aggregated candidate is

(13) $${\tilde{c}}_{\mathrm{set}}=\sum_{p}\alpha_{p}v_{p}.$$

The global forget and input gates are the means of the gates from the present perspectives:

(14) $$\begin{array}{cc} \bar{f} & ={\mathrm{mean}}_{p}f_{p}, \end{array}$$

(15) $$\begin{array}{cc} \bar{i} & ={\mathrm{mean}}_{p}i_{p}, \end{array}$$

(16) $$\begin{array}{cc} c_{t} & =\bar{f}c_{t-1}+\bar{i}{\tilde{c}}_{\mathrm{set}}. \end{array}$$

Finally, each perspective reads the joint memory through its output gate:

(17) $$\begin{array}{cc} h_{p} & =o_{p}\tanh\left(c_{t}\right), \end{array}$$

(18) $$\begin{array}{cc} h_{t} & ={\mathrm{mean}}_{p}h_{p}. \end{array}$$

Table 1 shows the dimensions and mask semantics used by the implemented cell. In the DSA experiments, H=64, F=9, and V=5; the learned *Q*, *K*, and *V* maps each operate from *H* to *H*, yielding one attention score per available view. The optional input cross-view attention module operates on the *F*-dimensional view features. It is enabled in the complete robust model and disabled only in the explicitly named no-attention ablation. These dimensions make explicit how the shared state and view-wise operations align in the implementation.

The forward pass first zero-fills masked inputs. Shared gate and candidate maps are evaluated at every position, but masked positions receive zero attention weight and are excluded from the masked means. The attention denominator therefore includes only available views. When all views are masked, the cell carries $\left(h_{t-1},c_{t-1}\right)$ forward. The time-reversed branch of the bidirectional encoder follows the same rule.

Table 2 illustrates the evaluation sequence from masked inputs through state updates, training, and test-time perturbations. It makes the handling of all-masked steps and the fixed subset and mask-trial protocol explicit, which supports reproduction of the reported comparisons. The prospective paired rerun reuses precomputed masks; historical results remain identified as non-paired.

**Permutation-invariance proposition.** Let π be any permutation of the *V* views, and let (πx,πm) denote the inputs and masks after the same permutation. Assume that view projections, candidate projections, and gate biases are shared across views, and that masked softmax assigns zero weight to unavailable views. For any recurrent state $\left(h_{t-1},c_{t-1}\right)$, the robust cell satisfies

(19) $$F\left(\pi x_{t},\pi m_{t},h_{t-1},c_{t-1}\right)=F\left(x_{t},m_{t},h_{t-1},c_{t-1}\right).$$

Shared gate maps give $i_{\pi (p)}=i_{p}$, $f_{\pi (p)}=f_{p}$, $o_{\pi (p)}=o_{p}$, and $c_{\pi (p)}^{\mathrm{local}}=c_{p}^{\mathrm{local}}$ after relabeling. The masked attention logits are therefore permuted, and softmax commutes with that permutation. The weighted sum $\sum_{p}\alpha_{p}v_{p}$ and the masked means of $f_{p}$, $i_{p}$, and $h_{p}$ remain unchanged. When all views are masked, carrying $\left(h_{t-1},c_{t-1}\right)$ forward is also invariant. Applying the equality at every time step proves invariance of the complete encoder by induction on *t*.

In the full model, view features pass through a lightweight cross-view attention module before entering the cell. This module is optional. The complete encoder is bidirectional [5]; its final forward and backward states are concatenated before the linear classifier.

**Figure 1:**
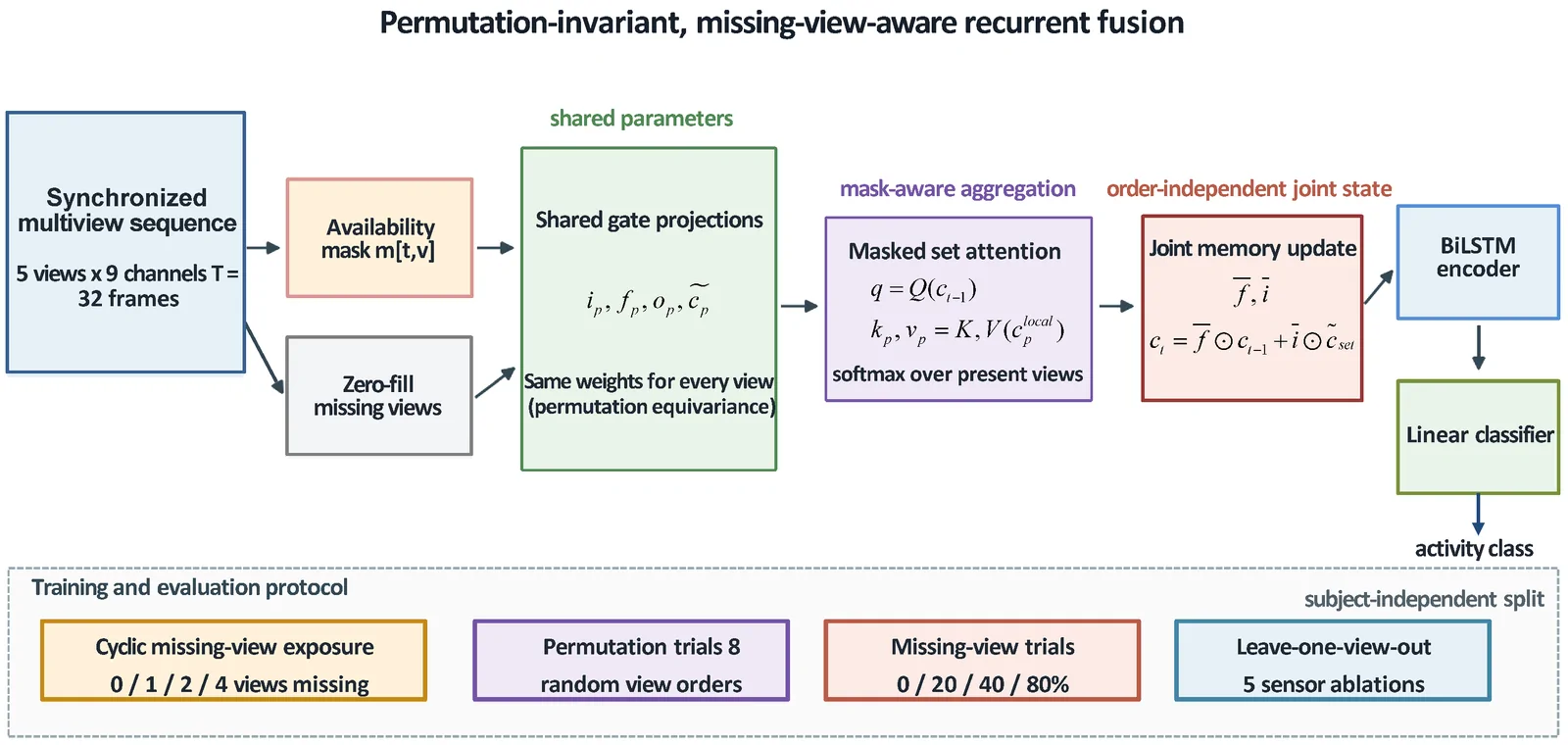
Model and evaluation overview.

Figure 1 illustrates the full processing path: five synchronized sensor views and an availability mask enter shared gate projections, masked set attention aggregates the view-level candidates, and the joint memory passes through a bidirectional encoder and linear classifier. The lower band shows cyclic missing-view exposure and the permutation, missing-view, and leave-one-view-out (LOO) evaluations. Sharing the projections and aggregating as a set makes the recurrent update insensitive to view indexing.

The robust cell is invariant to permutations of present views by construction. Shared projections and softmax aggregation over the view set produce the same output after the source order is permuted. The property follows from the update equations rather than from augmentation. Recent journal architectures use related permutation-invariant or permutation-aware representations [8,9,10].

### 3.4. Cyclic Missing-View Training Schedule

The initial experiments showed that the shared-parameter robust cell degrades when most views are missing. We therefore use a cyclic missing-view schedule to improve this behavior without adding source-specific parameters. During epoch *e* of *E* epochs, each training batch uses a target missing fraction

(20) $$r\left(e\right)=0.2+0.6\sin^{2}\;\left(\frac{\pi e}{E-1}\right).$$

With five views, the target fraction is rounded to an integer and capped so that at least one view remains. The schedule starts and ends with one missing view and reaches four missing views near the middle of training. Because it depends only on the number of available views, it preserves the inference-time permutation-invariance argument in Section 3.3. For scheduled models, the best checkpoint is selected by mean validation accuracy with zero, one, two, and four missing views rather than by full-view accuracy alone. The schedule is cyclic, not a monotonic easy-to-hard curriculum.

## 4. Experimental Setup

### 4.1. Dataset and Task

We use the UCI DSA dataset [7,24], which contains inertial and magnetic measurements from five Xsens MTx units attached to the torso, arms, and legs. Each unit contributes nine channels, comprising three accelerometer, three gyroscope, and three magnetometer measurements. Each archived sample is a 125-row by 45-channel matrix. We treat the five body-mounted units as synchronized perspectives. The main comparison uses the first ten activity classes, and the full extension uses all 19 available classes. Public body-worn and smartphone benchmarks use similar hardware layouts and split designs [29,30].

Each sample is uniformly downsampled to 32 frames. Sensor channels are normalized independently using training-set statistics. We use a subject-independent split with subjects 1–4 for training, subjects 5–6 for validation, and subjects 7–8 for testing. This protocol avoids the optimistic bias that arises when examples from the same subject appear in both training and test sets [30,31].

### 4.2. Models and Training

All sequence models share the following configuration unless noted:

- Hidden size: 64;
- Input dimension per perspective: 9;
- Five perspectives;
- Batch size: 128;
- Optimizer: AdamW;
- Learning rate: $10^{-3}$, cosine decay to $10^{-5}$;
- Weight decay: $10^{-4}$;
- Epochs: 15;
- Dropout on the classification head: 0.2;
- Ten model-initialization/training seeds: 0–9 for the revision matrix.

We use AdamW with a cosine learning-rate schedule and apply dropout to the classification head. During training, each fixed-dropout model follows the same augmentation rule, removing exactly one of five views from every training example. Because data-loader random states can diverge after initialization, comparable models are not guaranteed to receive identical sample–mask pairs. The checkpoint with the highest full-view validation accuracy is selected for testing. Scheduled models follow Section 3.4 and are selected by mean validation accuracy with 0, 1, 2, and 4 missing views. These are the procedures used for the main reported historical results.

For the prospective paired rerun, masks are materialized before training and reused across architectures within each seed, batch, epoch, and missing-view condition. This paired protocol is a registered follow-up design and is distinct from the main historical results reported later in this section, which retain the original per-loader random-mask procedure. Those results are therefore not presented as results from a fully paired masking experiment.

The revision matrix also separates shared projection, set attention, input cross-view attention, candidate aggregation, and missing-view scheduling. The baselines are:

- Concat + BiLSTM: concatenates the five nine-dimensional source vectors at each time step and adds a per-source availability indicator.
- Per-view BiLSTM: trains one bidirectional LSTM per source and averages the present source states.
- Generalized chain comparator: the generalized paper-inspired chain model described in Section 3.2.
- Chain comparator + view permutation: the same architecture trained with random view permutations. This represents a data-augmentation attempt to reduce order sensitivity.
- Robust MP-LSTM (ours): the proposed shared-projection set-attention cell.
- Robust MP-LSTM + cyclic schedule: the same robust cell trained with cyclic missing-view exposure and missing-aware validation.
- Masked-mean + BiLSTM, shared-set mean + BiLSTM, shared-set attention + BiLSTM, and Set Transformer + BiLSTM.
- Robust MP-LSTM with mean candidate aggregation, with and without input cross-view attention, to isolate the two attention paths.

### 4.3. Evaluation

The 10-class test set contains 1,200 segments, and the full 19-class test set contains 2280 segments from subjects 7 and 8. Revision full-view accuracies are reported as the mean, sample SD, and two-sided 95% *t* interval across ten training seeds. These intervals describe optimization variability only because the segments are repeated observations from two held-out people, not independent draws from a target population. A paired two-sided *t* test is used only for matched-seed descriptive comparisons and is not interpreted as a population-level result.

Order-perturbation, missing-view, and leave-one-view experiments use a fixed 720-segment subset drawn without replacement from the test set with NumPy random seed 2026 and the seed-0 model for each architecture. The 10-class subset contains 67–79 segments per class. We measure order robustness in eight trials, assigning each segment a random permutation of the five streams in each trial. We measure missing-view robustness in eight trials with exactly 0, 1, 2, or 4 unavailable views per segment, corresponding to missing fractions of 0%, 20%, 40%, and 80%. LOO accuracy is measured by removing each body location in turn. The SD across mask or permutation trials captures perturbation variability conditional on one trained model and one subset, not training or sampling uncertainty.

Accuracy is the primary metric. The dataset is class-balanced before subsetting. A separate audit script verifies each saved accuracy against sample-level predictions and exports overall and per-subject macro-F1 values. All seed-level statistics were computed from the saved per-seed test accuracies by an independent analysis script. We do not present a segment-level bootstrap as a population interval because only two test subjects are held out.

The paired design is registered as a one-factor-at-a-time comparison. Architecture changes use the same precomputed masks and full-view checkpoint rule; masking-policy changes use the same architecture and checkpoint rule; and checkpoint-criterion changes use the same architecture and mask schedule. The generator and configuration files are provided for this rerun. Historical three-seed results remain identified as pre-registration results.

### 4.4. Efficiency and Deployment Protocol

For each architecture, we measured trainable parameters, forward latency, throughput, and peak allocated CUDA memory on the same RTX 5090 system after 20 warm-up iterations and 100 timed repetitions. The measurements used batch sizes 1 and 128 with the DSA input shape (T,V,F)=(32,5,9). The latency was the wall-clock time for the complete forward pass, including masking and classification. We reported parameter counts and wall-clock measurements rather than assigning a synthetic FLOP count to fused recurrent kernels.

### 4.5. Independent-Dataset Attempt and Scope

We attempted to add the public UCI Human Activity Recognition Using Smartphones dataset using its official train/test split and a fixed subject-level validation subset. The official archive could not be obtained as a complete, checksum-valid ZIP in this execution environment after repeated transfers. We therefore report no UCI-HAR accuracy.

## 5. Results

### 5.1. Full-View Test Accuracy

Table 3 shows the mean full-view accuracy over three seeds, as well as the SD, parameter count, and training time per seed. Relative to the generalized chain comparator, the robust cell uses 66.0% fewer parameters (65,534 versus 192,906) and has a 41.5% shorter recorded training time (96.4 s versus 164.9 s). The timing difference is partly implementation-dependent because the comparator loops over views in Python 3.12.8. The mean accuracy is 3.53 percentage points below the chain comparator and 4.89 points below early concatenation; the robust model also varies more across the three seeds, so its SD is imprecisely estimated. The lower cost is consistent with shared projections replacing position-specific parameters, while the accuracy difference shows the trade-off associated with this parameter sharing.

**Figure 2:**
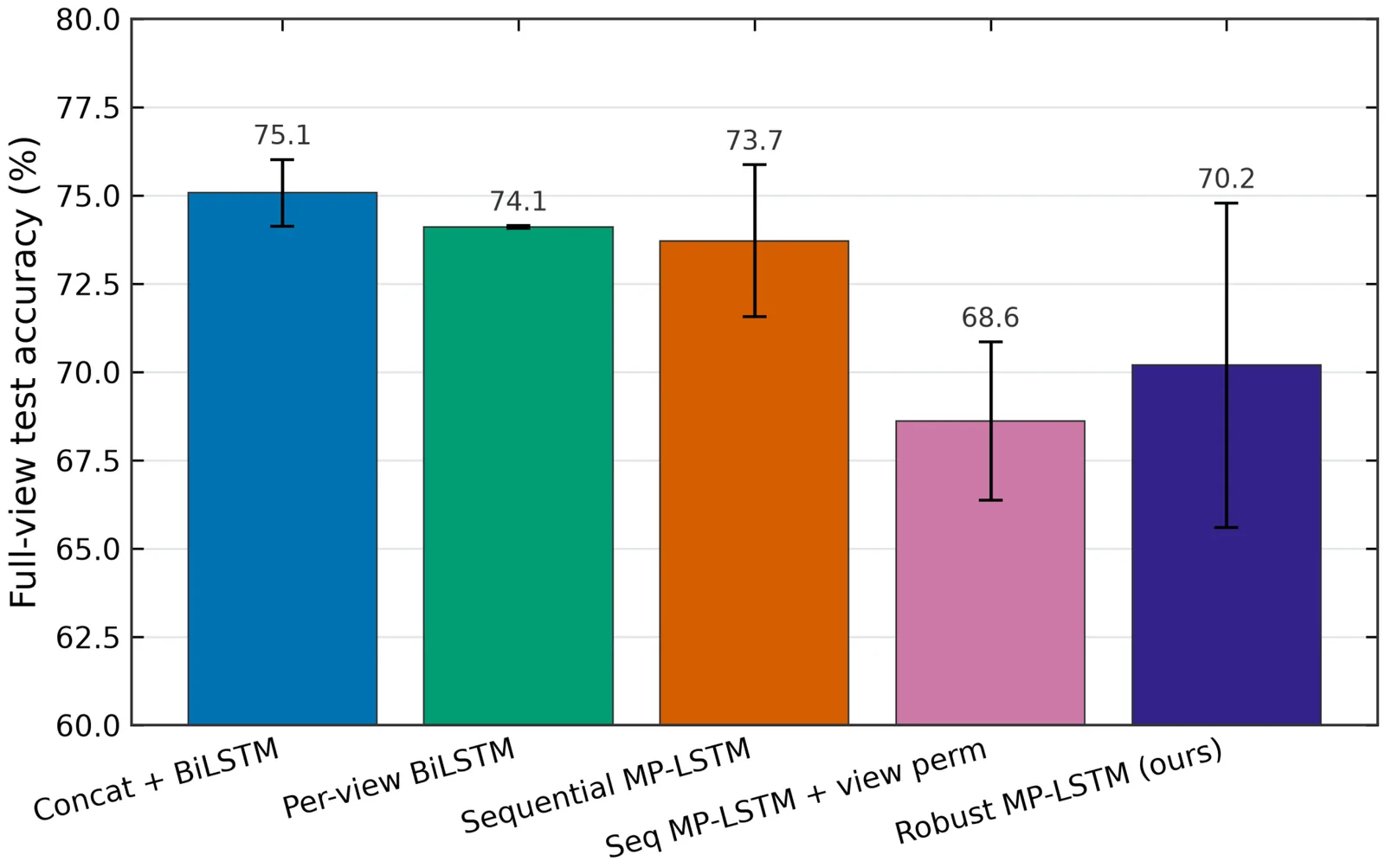
Full-view accuracy across seeds.

Figure 2 shows that the robust cell’s mean full-view accuracy is below the chain and concatenation baselines, with a wider seed-level spread. This pattern matches Table 3 and is consistent with the shared cell trading source-specific capacity for a smaller, order-invariant update.

### 5.2. Accuracy–Efficiency and Component Ablation

The revision matrix independently evaluates shared projection, candidate aggregation, input cross-view attention, and cyclic missing-view training, with masking strategy and checkpoint-selection criteria assessed in separate protocol conditions. The current design does not fully isolate interactions among shared projection, candidate aggregation, input attention, masking, optimization, and checkpoint selection. The proposed configuration reached 69.66% mean accuracy (SD 3.85%) with 65,534 trainable parameters. Removing input cross-view attention reduced the mean to 69.47%, while replacing attention-based candidate aggregation with a mean reduced it to 66.87%; these differences are descriptive and are not interpreted as isolated causal effects. On the same RTX 5090 system, the proposed cell had a median batch-one forward latency of 114.74 ms per sample and a throughput of 8.62 samples s^−1^, compared with 181.75 ms and 5.50 samples s^−1^ for the sequential comparator. At batch size 128, the corresponding per-sample latencies were 0.999 and 1.471 ms. These measurements include masking and classification and quantify forward inference, whereas the earlier benchmark reports training time per seed. We also profiled the Set Transformer + BiLSTM implementation for efficiency, but its full ten-seed accuracy protocol was not completed; it is therefore included as an implementation status check, not as a fully validated quantitative state-of-the-art comparison.

**Figure 3:**
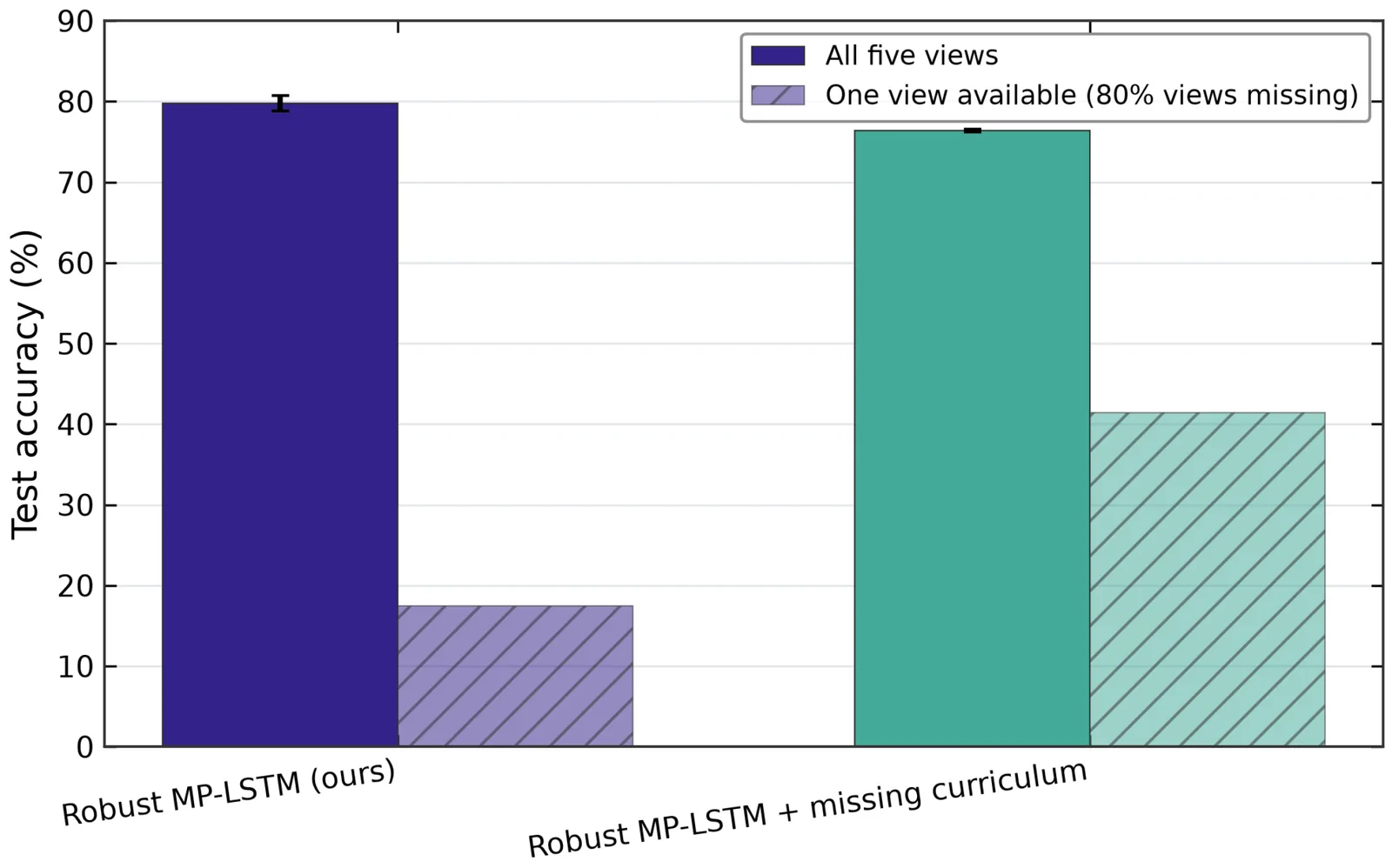
Accuracy–efficiency trade-off. Green bars denote the missing-view curriculum variant; hatched bars denote the one-view-available condition.

Figure 3 shows the ten-seed accuracy–latency trade-off: the robust MP-LSTM combines a median batch-one latency of 114.74 ms with mean accuracy of 69.66%, while the sequential comparator is slower at 181.75 ms. The error bars represent seed-level accuracy SD. The shorter latency is consistent with eliminating the comparator’s sequential view loop; the accuracy remains lower than several full-view baselines, so the figure presents a cost–performance trade-off rather than a general ranking. Measurements followed 20 warm-up iterations and 100 timed repetitions on the same RTX 5090 system.

### 5.3. View-Order Robustness

Table 4 shows fixed-order and permuted-order accuracy on the 720-segment subset. Reassigning source tensors to different view positions lowers chain-comparator accuracy by 51.72 percentage points. Training that comparator with random permutations recovers part of this robustness, while reducing fixed-order accuracy to 65.56% and leaving a permutation-trial SD of 1.03 points. The robust cell remains at 75.14% with an SD of 0.00 because view order does not enter its deterministic update. This test measures index-order sensitivity, not physical sensor relocation or changes in channel semantics.

**Figure 4:**
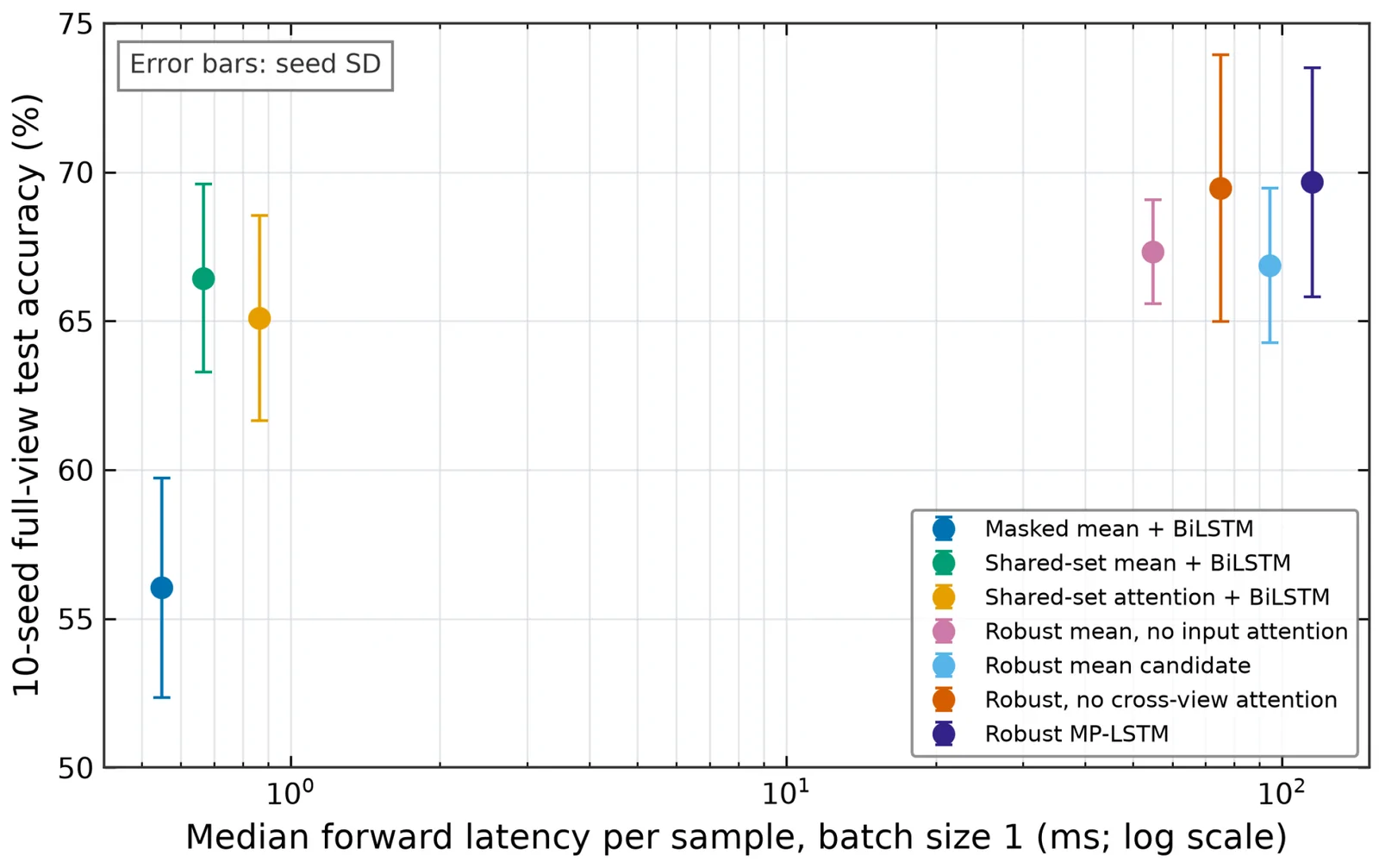
Accuracy under view permutation.

Figure 4 shows the same permutation response graphically: the robust cell’s accuracy is unchanged across view orders, whereas the unaugmented chain comparator drops sharply. The contrast follows directly from the robust cell’s shared projections and symmetric aggregation; augmentation reduces, but does not remove, the comparator’s order sensitivity. Error bars show trial SD, not uncertainty over models or subjects.

### 5.4. Missing-View Robustness

Table 5 shows mean accuracy over eight random missing-view masks for one seed-0 model. With one view missing, the robust cell loses 5.61 percentage points from its full-view accuracy; with two missing, it retains 60.36%, or 80.3% of full-view accuracy. With four of five views missing, accuracy falls to 36.65%, above the 10.0% random-chance level for a balanced 10-class task but below the sequential baseline. This decline is consistent with the smaller set of sensor evidence available to the shared aggregation.

The chain comparator is more stable under missing views in this benchmark. Shared parameters enforce order invariance, but the resulting cell retains less source-specific dynamics when several sources disappear. This observation links architecture and performance without isolating a causal mechanism.

**Figure 5:**
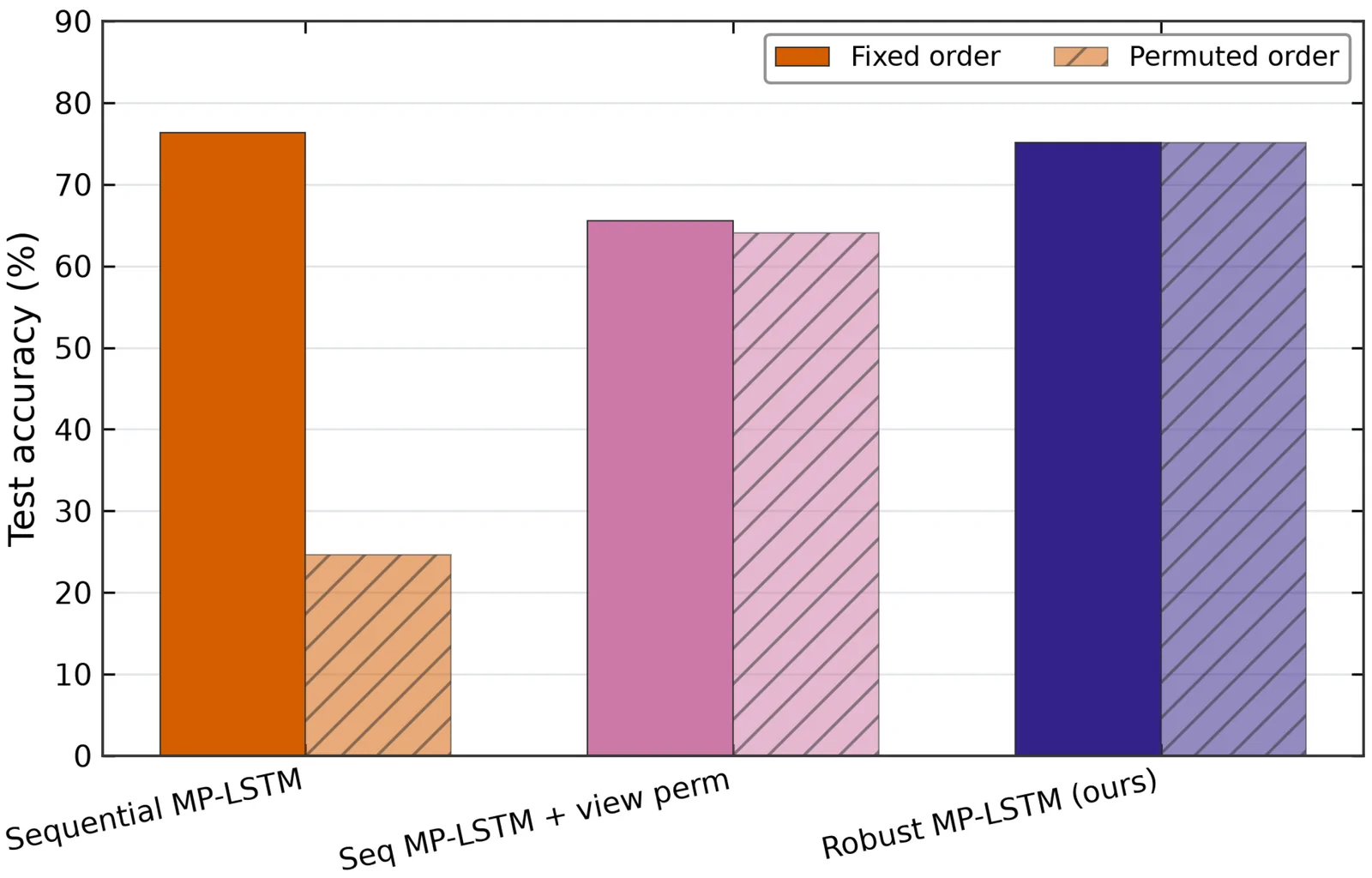
Missing-view accuracy. Orange, pink, and purple denote the sequential, permutation-augmented sequential, and robust MP-LSTM models, respectively; hatched bars denote permuted-order accuracy.

Figure 5 shows accuracy decreasing as the missing-view rate rises, with the strongest decline for the robust cell at four missing views. This trend is expected when fewer sensor streams remain to represent the activity and is consistent with the larger performance gap under severe dropout in Table 5.

### 5.5. LOO Interpretability

Table 6 shows accuracy after removing one body-mounted sensor. The robust cell has a mean leave-one-view accuracy of 69.25%, 1.08 percentage points below the chain comparator. Its descriptive range across removed sensors is 7.50 percentage points compared with 11.94 for the comparator. The robust cell’s lowest result is 64.03% with the torso removed; the comparator’s lowest is 62.36% with the left leg removed. These five correlated ablations do not establish more statistically balanced source dependence.

**Figure 6:**
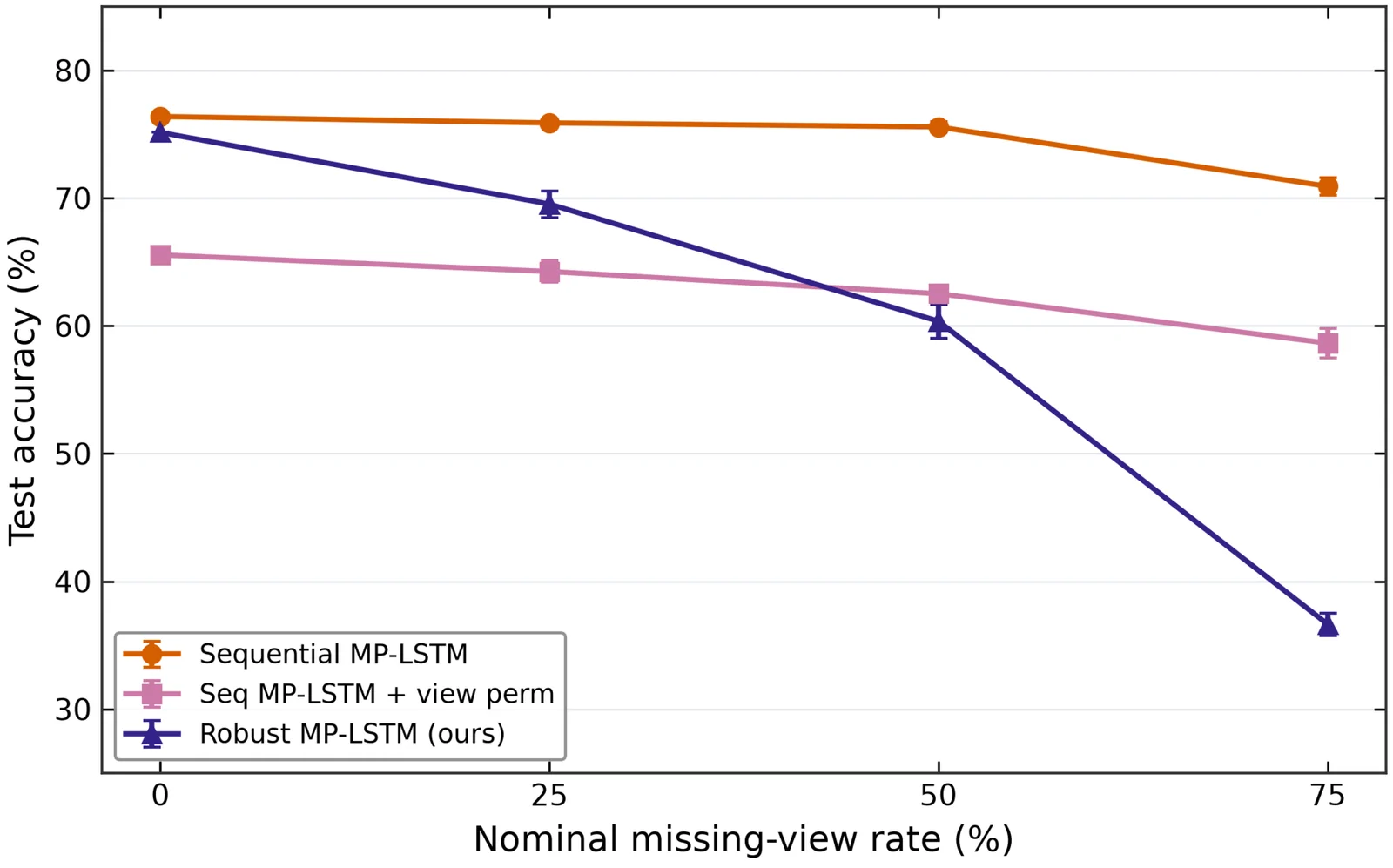
Leave-one-view-out profiles.

Figure 6 shows the sensor-specific profiles summarized in Table 6. The generalized chain comparator fluctuates more across the five removed-sensor conditions, whereas both robust variants remain within narrower ranges, with the cyclic schedule producing the most even profile. This pattern is consistent with reduced dependence on any single view under shared updates, but the five correlated removals do not establish statistically balanced source importance.

### 5.6. Cyclic Missing-View Schedule and 19-Class Extension

Table 7 shows how cyclic missing-view training changes the 10-class results: four-missing-view accuracy rises from 36.65% to 49.31%, while three-seed mean full-view accuracy falls from 70.19% to 67.67%. Exposing the model to a changing missing-view count during training improves its severe-dropout response, with a corresponding full-view trade-off.

Table 8 shows the schedule’s effect across all 19 activity classes. Four-missing-view accuracy rises from 17.47% to 41.41%, while three-seed mean full-view accuracy falls by 3.40 percentage points; the scheduled model nevertheless remains below the chain comparator under severe dropout. In a single-seed contextual run, the comparator retained 67.74% with one view available, versus 41.41% for the scheduled model. On the same subset, their full-view accuracies were 74.72% and 76.11%, respectively, and their permuted-order means were 21.37% and 76.11%. The comparator has one 19-class training run, so this is a contextual, not multi-seed, comparison. The pattern is consistent with cyclic training allocating more exposure to sparse-view inputs, at the cost of full-view performance.

We also tested an availability-conditioned robust cell whose global gate bias and attention query depend on the number of present views. Although this conditioning is order-invariant, combining it with the cyclic schedule did not improve full-view or severe-dropout accuracy, so it is not included in the final model.

**Figure 7:**
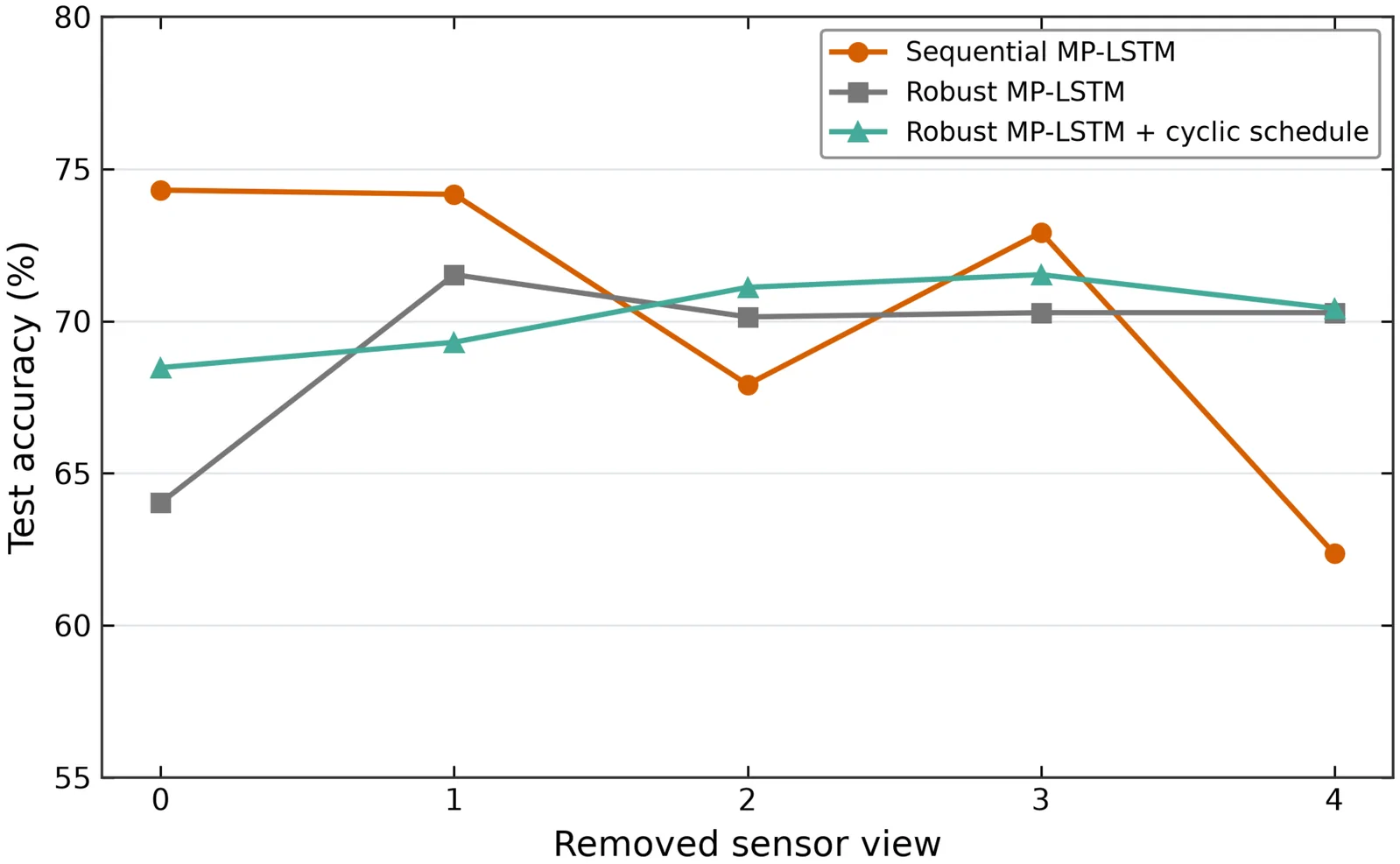
Cyclic schedule: missing-view and LOO results.

Figure 7 shows that the cyclic schedule improves the 10-class model’s severe-missing-view accuracy and narrows the observed LOO range. The error bars in the missing-view panel represent variation across eight mask trials. The gains are consistent with training on a broader range of view availability.

**Figure 8:**
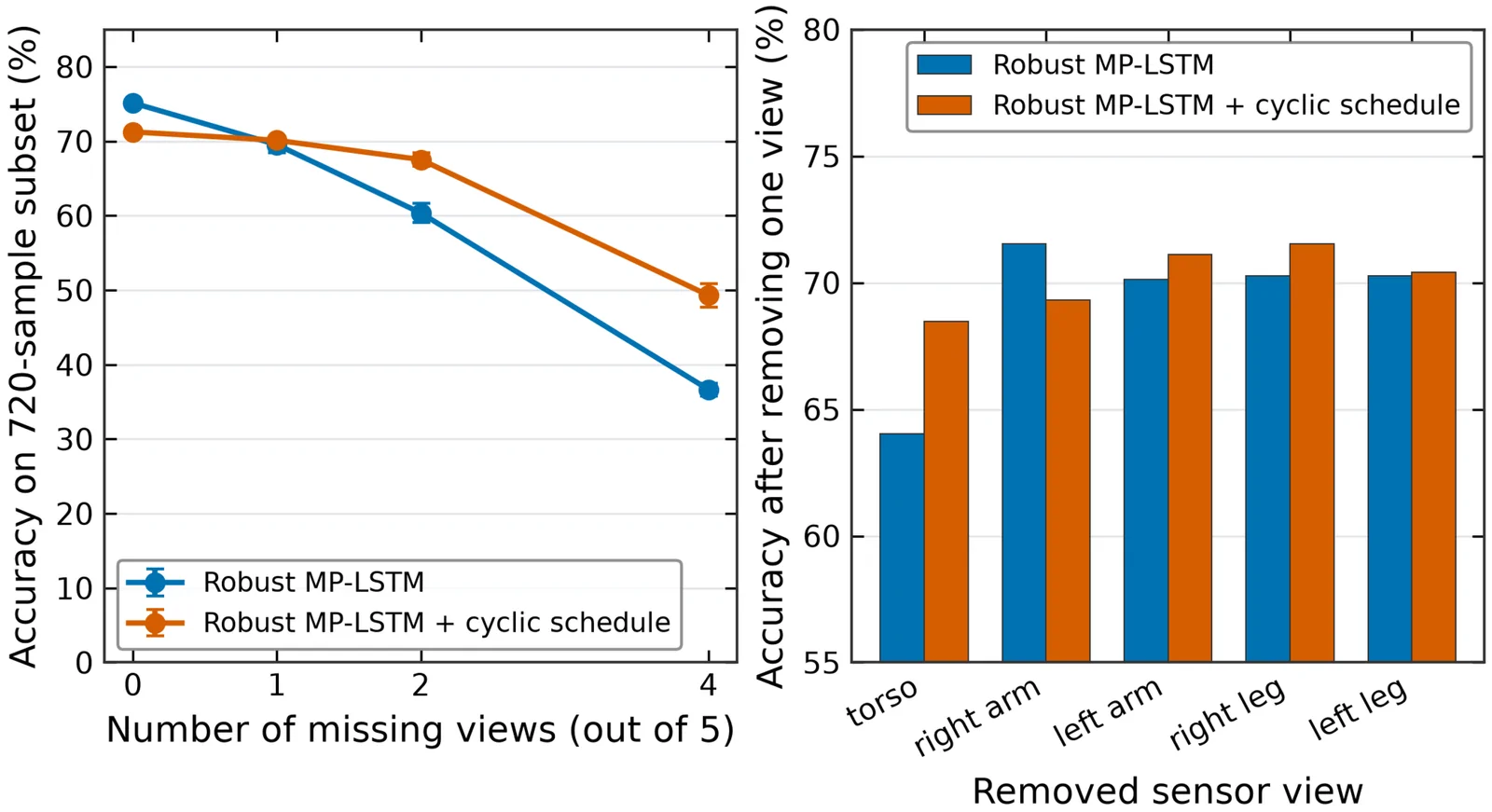
The 19-class accuracy under view removal.

Figure 8 shows full-view and one-available-view accuracy in the 19-class contextual run. The scheduled model retains less accuracy when only one view remains than the chain comparator, despite its improvement over the unscheduled robust model. This contrast shows that gains from the schedule are conditional on the baseline and missing-view severity.

### 5.7. Subject-Level and Class-Balanced Diagnostics

Because the test set contains two independent participants, we report subject-level accuracy and macro-F1 alongside aggregate accuracy. In the 10-class task, the three-seed subject means for the generalized chain comparator were 84.11%/81.03% accuracy and macro-F1 on subject 7, and 64.00%/60.12% on subject 8; the robust cell gave 74.50%/69.06% and 65.89%/60.60%, respectively. In the 19-class extension, the robust full-view seed-0 model achieved 79.50%/76.45% on subject 7 and 80.03%/77.33% on subject 8, whereas the cyclic model achieved 74.62%/71.72% and 78.13%/75.52%. These diagnostics make subject-specific variation visible alongside aggregate results.

Table 9 shows that the relative accuracy and macro-F1 of the chain comparator and robust cell differ between subjects 7 and 8. The subject-level contrast explains why aggregate scores alone can obscure participant-specific performance; these values are descriptive for the two held-out participants.

Table 10 shows the full-view accuracy and macro-F1 of the two scheduled and unscheduled models separately for subjects 7 and 8. The differences between participants remain visible in the 19-class extension, so these seed-0 diagnostics complement rather than replace population-level evaluation.

**Figure 9:**
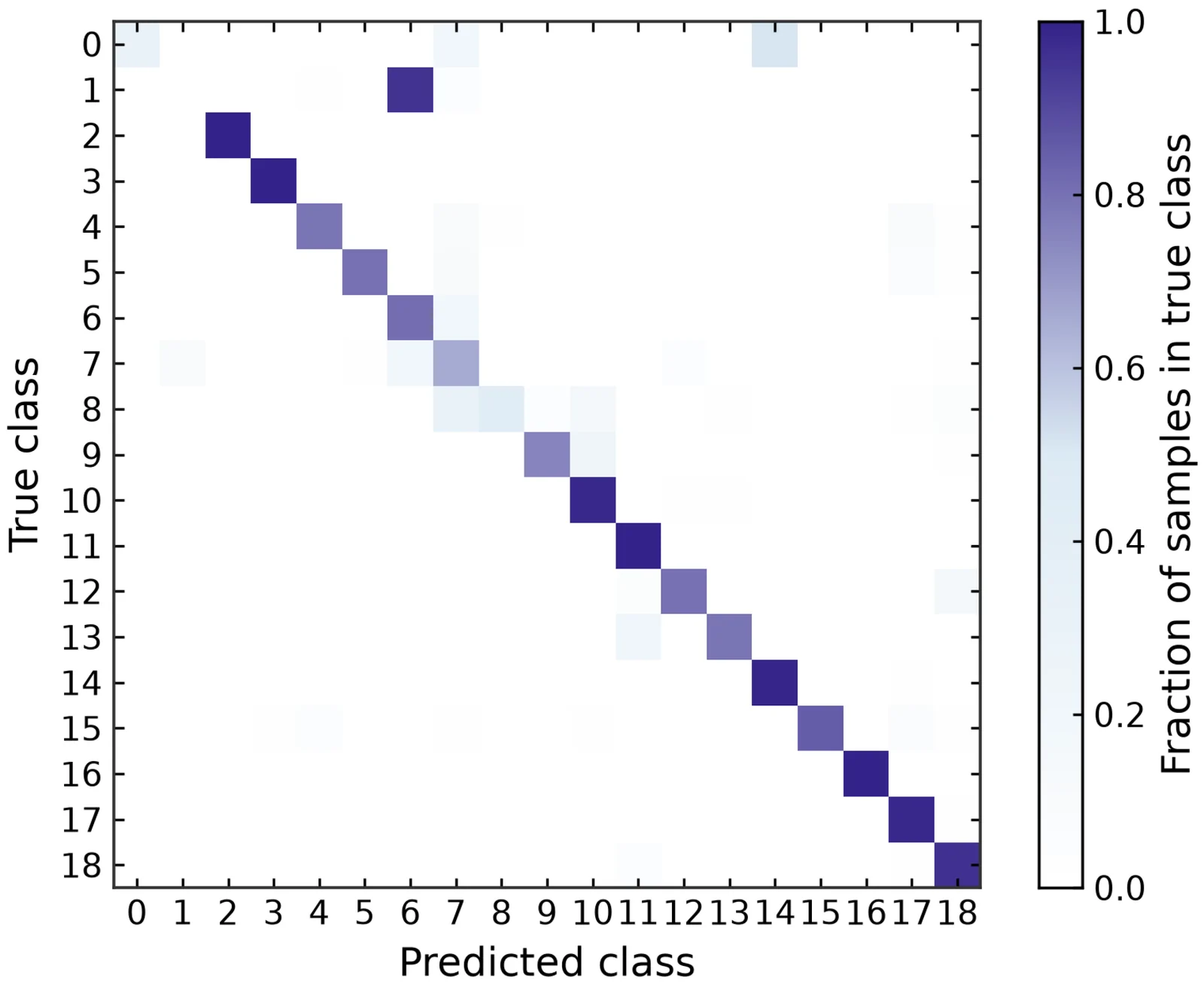
19-class confusion matrix.

Figure 9 shows the class-wise distribution of full-view predictions for the robust MP-LSTM seed-0 model. Counts concentrate on the diagonal for most classes, while larger off-diagonal cells include class 0 predicted as class 14 (61 samples), class 8 as class 7 (35), and class 9 as class 10 (29). Row-normalized colour makes these patterns comparable across true classes; the remaining confusions are consistent with class overlap in the sensor representations, although this matrix alone cannot identify their cause. The stored severe-missing-view results contain aggregate trial accuracies rather than per-sample predictions, so an equivalent confusion matrix is unavailable. Macro-F1 and subject-level diagnostics are available for the saved full-view predictions. The severe-missing-view experiments report eight-mask aggregate accuracies and trial SDs.

## 6. Discussion

The experiments distinguish three forms of robustness. First, the proposed deterministic evaluation-time mapping is permutation-invariant by construction. The augmentation-trained chain comparator remains usable after permutation, but its output changes with the reassignment and its fixed-order accuracy decreases. Second, all evaluated models accept a static source mask. The robust cell trained with one-view removal degrades faster than the chain comparator when two or more sources are removed, while the cyclic schedule recovers part of that gap. Third, LOO analysis identifies which removal is most damaging for each trained model. The robust cell has a smaller observed range, a pattern that is consistent with more even source dependence but not sufficient to establish it.

The measured trade-off is consistent with the design. Shared projections remove position-specific parameters that a chain model can use for specialization. Optimization, cross-view attention, parameter count, and comparator implementation also differ and were not isolated in a complete ablation. When source identities are stable and informative, an order-sensitive model may be preferable; the proposed architecture is intended for systems in which input order is arbitrary or unstable at deployment.

## 7. Limitations and Future Work

The study uses one dataset, one fixed subject split, and two held-out test participants. The results therefore describe this controlled case study rather than a general population. The many segments per participant do not replace independent subjects, and three training seeds characterize optimization variability rather than population uncertainty. The 720-segment perturbation subset is mildly class imbalanced, and all perturbation results use seed 0. The 19-class chain comparator has one training run. In the 10-class task, the robust model has lower mean full-view accuracy and greater optimization variability; after scheduling, the severe missing-view performance remains below that of the chain comparator. The ablation matrix assesses selected components under controlled one-factor-at-a-time conditions, but it does not identify all component interactions. The Set Transformer comparison is incomplete and should not be interpreted as a fully validated quantitative benchmark. The main reported masking results are historical non-paired evaluations, whereas the paired protocol is prospective. Severe-missing-view analyses report aggregate accuracy and trial SDs, but corresponding class-level predictions were not retained; per-subject and macro-F1 diagnostics are available for the saved full-view predictions. Future work should use participant-level resampling or repeated subject-held-out folds, at least 10 training seeds for optimization comparisons, a faithful original-code comparator, component ablations, calibrated uncertainty, and an independent dataset.

The study is therefore a controlled descriptive case study of one DSA split with two held-out participants. The subject-level tables show substantial differences between these two held-out participants. Class-level confusion matrices are available for saved full-view predictions, whereas severe-missing-view class-level metrics were not retained by the historical aggregate evaluator and require a paired rerun. The severe-missing-view results are therefore limited to aggregate accuracy and trial SDs.

Future work should evaluate the robust cell on longer video sequences, richer visual features, asynchronous sources, different frame rates, and real camera-dropping scenarios. A useful architectural extension would share only the memory-aggregation stage while retaining optional source-specific input encoders when source identity is informative. Source-level reconstruction losses, asynchronous test-time inference, and availability-conditioned gate biases are additional directions; the current implementation already accepts arbitrary view counts and masks.

## 8. Conclusions

We implemented a permutation-invariant, missing-view-aware recurrent fusion cell and evaluated it in a controlled case study using one fixed DSA split with subjects 7 and 8 as the held-out test participants. At deterministic inference, the cell is exactly invariant to view permutation and is smaller than the generalized chain comparator. A cyclic missing-view schedule improves seed-0 four-missing-view accuracy by 12.66 percentage points in the 10-class task and 23.94 points in the 19-class task, while three-seed mean full-view accuracy decreases by 2.53 and 3.40 points, respectively. These findings establish the implemented invariance property and quantify a measurable but incomplete recovery of severe missing-view performance after cyclic training. The gains are accompanied by lower full-view accuracy on the evaluated split, and the generalized chain comparator remains stronger under severe dropout. The ablation, paired-masking, Transformer, and class-level severe-missing results remain bounded by the protocol limitations described above; broader generalization requires additional datasets and independent held-out participants.

## Figures and Tables

**Table 1:** Cell dimensions and masks.

Quantity	Dimension	Role
$x_{t}$	V×F=(5,9)	synchronized view features at time *t*
$h_{t-1},c_{t-1}$	H=64	previous recurrent hidden and cell states
$i_{p},f_{p},o_{p},{\tilde{c}}_{p}$	*H*	shared gate and candidate vectors
$c_{p}^{\mathrm{local}}$	*H*	per-view candidate memory
$Q\left(c_{t-1}\right),K\left(c_{p}^{\mathrm{local}}\right),V\left(c_{p}^{\mathrm{local}}\right)$	*H*	query, key, and value projections
$\alpha_{p}$	scalar	masked softmax weight for available view *p*
$m_{t}$	*V*	Boolean mask; 1 means unavailable
$h_{t},c_{t}$	*H*	updated recurrent states

**Table 2:** Evaluation pseudocode. Bold labels identify the forward, training, and test-perturbation stages.

Procedure
**Forward:** for t=1,…,T, select $m_{t}$, zero-fill unavailable $x_{t,p}$, optionally apply cross-view attention with the same mask, compute shared gates and local candidates, apply masked softmax to available candidates, update $\left(h_{t},c_{t}\right)$, and carry the previous state if all views are masked.
**Training:** initialize the model with seed *s*; for each epoch, draw a precomputed batch-mask schedule from the declared seed; optimize cross-entropy with AdamW; select either full-view validation or the prespecified mean over 0/1/2/4 missing views, according to the registered condition.
**Test perturbation:** draw the fixed 720-segment subset with NumPy seed 2026; for each of eight trials and each missing-view count 0, 1, 2, or 4, generate sample-wise masks by seeded random ranking; report mean and trial SD.

**Table 3:** Full-view accuracy and model cost.

Method	Mean Accuracy (%)	Std (%)	Parameters	Training Time/Seed (s)
Concat + BiLSTM	75.08	0.94	60,938	2.2
Per-view BiLSTM	74.11	0.04	196,106	4.0
Generalized chain comparator	73.72	2.15	192,906	164.9
Chain comparator + view perm	68.61	2.24	192,906	168.6
Robust MP-LSTM (ours)	70.19	4.59	65,534	96.4

**Table 4:** Accuracy under view permutation.

Method	Fixed Order (%)	Permuted Order (%)	Permutation Std (%)
Generalized chain comparator	76.39	24.67	1.07
Chain comparator + view perm	65.56	64.03	1.03
Robust MP-LSTM (ours)	75.14	75.14	0.00

**Table 5:** Accuracy under missing-view masks.

Method	0 Views Missing (%)	1 View Missing (%)	2 Views Missing (%)	4 Views Missing (%)
Generalized chain comparator	76.39	75.89	75.57	70.92
Chain comparator + view perm	65.56	64.25	62.52	58.65
Robust MP-LSTM (ours)	75.14	69.53	60.36	36.65

**Table 6:** Leave-one-view-out accuracy.

Removed View	Chain Comparator (%)	Chain + View Perm (%)	Robust MP-LSTM (%)
Torso	74.31	60.14	64.03
Right arm	74.17	69.03	71.53
Left arm	67.92	62.08	70.14
Right leg	72.92	61.81	70.28
Left leg	62.36	42.08	70.28
Mean	70.33	59.03	69.25
Range	11.94	26.94	7.50

**Table 7:** Cyclic-schedule results: 10-class task.

Method	Full-View Test Mean %	Std %	4/5 Views Missing %	LOO Mean %	LOO Range %
Robust MP-LSTM	70.19	4.59	36.65	69.25	7.50
Robust MP-LSTM + cyclic schedule	67.67	4.70	49.31	70.17	3.06

**Table 8:** Cyclic-schedule results: 19-class task.

Method	Full-View Test Mean %	Std %	4/5 Views Missing %	LOO Mean %	LOO Range %
Robust MP-LSTM	79.77	0.92	17.47	73.14	13.47
Robust MP-LSTM + cyclic schedule	76.37	0.16	41.41	74.22	8.06

**Table 9:** Subject-level diagnostics: 10-class task.

Method	Subject 7 Accuracy	Subject 7 Macro-F1	Subject 8 Accuracy	Subject 8 Macro-F1
Generalized chain comparator	84.11	81.03	64.00	60.12
Robust MP-LSTM	74.50	69.06	65.89	60.60
Robust MP-LSTM + cyclic schedule	66.39	62.53	68.94	63.88

**Table 10:** Subject-level diagnostics: 19-class task.

Method	Subject 7 Accuracy	Subject 7 Macro-F1	Subject 8 Accuracy	Subject 8 Macro-F1
Robust MP-LSTM	79.50	76.45	80.03	77.33
Robust MP-LSTM + cyclic schedule	74.62	71.72	78.13	75.52

## Data Availability

The data presented in this study are available in the UCI Machine Learning Repository at https://archive.ics.uci.edu/dataset/256/daily+and+sports+activities (accessed on 3 October 2026), reference number [25]. These data were derived from the following resources available in the public domain: the UCI Daily and Sports Activities dataset (https://archive.ics.uci.edu/dataset/256/daily+and+sports+activities (accessed on 3 October 2026)). The split definition, fixed 720-segment indices, mask/permutation protocol, audit/export script, README, and links to the existing loaders, training scripts, saved prediction audits, revision matrix, and efficiency measurements presented in this study are included in the Supplementary Materials.

## Supplementary Materials

The following supporting information can be downloaded at https://www.mdpi.com/article/10.3390/s26196330/s1: split definition, fixed 720-segment subset indices, mask/permutation protocol, audit/export scripts, saved prediction summaries, revision matrix, and efficiency measurements.

## References

[1] Dimitri G.M. (2022). A short survey on deep learning for multimodal integration: Applications, future perspectives and challenges. Computers.

[2] Bayoudh K. (2024). A survey of multimodal hybrid deep learning for computer vision: Architectures, applications, trends, and challenges. Inf. Fusion.

[3] Abdu S.A., Yousef A.H., Salem A. (2021). Multimodal video sentiment analysis using deep learning approaches: A survey. Inf. Fusion.

[4] Vidya B., Sasikumar P. (2022). Wearable multi-sensor data fusion approach for human activity recognition using machine learning algorithms. Sens. Actuators A Phys..

[5] El-Adawi E., Essa E., Handosa M., Elmougy S. (2024). Wireless body area sensor networks based human activity recognition using deep learning. Sci. Rep..

[6] Balaha H.M., Hassan A.H. (2023). Comprehensive machine and deep learning analysis of sensor-based human activity recognition. Neural Comput. Appl..

[7] Wang J., Liu Y., Pei J., Huang Z., Zhang X., Yang H. (2021). Multiview attention CNN-LSTM network for SAR automatic target recognition. IEEE J. Sel. Top. Appl. Earth Obs. Remote Sens..

[8] Chen Y., Ye Y. (2023). Neural decoders with permutation invariant structure. J. Franklin Inst..

[9] Deng C., Deng L., Yang C. (2024). Random permutation set reasoning. IEEE Trans. Pattern Anal. Mach. Intell..

[10] Pérez-Mon M., Moreo F., del Coz J.J., González J.A. (2024). Quantification using permutation-invariant networks based on histograms. Neural Comput. Appl..

[11] Nafea I., Abdul W., Muhammad G., Alsulaiman M. (2021). Sensor-based human activity recognition with spatio-temporal deep learning. Sensors.

[12] Gupta P. (2021). Deep learning based human activity recognition (HAR) using wearable sensor data. Int. J. Inf. Manag. Data Insights.

[13] Gao W., Zhang L., Teng Q., He J., Wu H. (2021). DanHAR: Dual attention network for multimodal human activity recognition using wearable sensors. Appl. Soft Comput..

[14] Xu C., Zhang L., Huang C., Wu J., Song Y. (2022). Deformable convolutional networks for multimodal human activity recognition using wearable sensors. IEEE Trans. Instrum. Meas..

[15] Vuong T.P., Doan T.S., Takasu A. (2023). Deep wavelet convolutional neural networks for multimodal human activity recognition using wearable inertial sensors. Sensors.

[16] Zhou X., Zhao X., Liu Y., Lu H., An Y., Liu Y. (2023). Multi-sensor data fusion and CNN-LSTM model for human activity recognition system. Sensors.

[17] Liu Y., Gao H., Li Y., Liu J. (2024). Human activity recognition through deep learning: Leveraging unique and common feature fusion in wearable multi-sensor systems. Appl. Soft Comput..

[18] Wang Y., Kang J. (2025). Adaptive temporal attention mechanism and hybrid deep CNN model for wearable sensor-based human activity recognition. Sci. Rep..

[19] Ciliberto M., Fortes Rey V., Calatroni A., Lukowicz P., Roggen D. (2021). Opportunity++: A multimodal dataset for video- and wearable, object and ambient sensors-based human activity recognition. Front. Comput. Sci..

[20] Hochreiter S., Schmidhuber J. (1997). Long short-term memory. Neural Comput..

[21] Gers F.A., Schmidhuber J., Cummins F. (2000). Learning to forget: Continual prediction with LSTM. Neural Comput..

[22] Graves A., Schmidhuber J. (2005). Framewise phoneme classification with bidirectional LSTM and other neural network architectures. Neural Netw..

[23] Zhong Y., Liu Y. (2025). Efficient human activity recognition using machine learning and wearable sensor data. Appl. Sci..

[24] Narkhede P., Walambe R., Poddar S., Kotecha K. (2021). Incremental learning of LSTM framework for sensor fusion in attitude estimation. PeerJ Comput. Sci..

[25] Altun K., Barshan B., Tunçel O. (2010). Comparative study on classifying human activities with miniature inertial and magnetic sensors. Pattern Recognit..

[26] Tokas P., Semwal V.B., Jain S. (2025). A lightweight and explainable hybrid deep learning model for wearable sensor-based human activity recognition. IEEE Sens. J..

[27] Guo Y., Nakayama K. (2025). Towards user-generalizable wearable-sensor-based human activity recognition: A multi-task contrastive learning approach. Sensors.

[28] Guo X., Yang M., Lu G.-F. (2025). Tensor-based late fusion incomplete multiview clustering. IEEE Trans. Multimed..

[29] Qian T., Wang J. (2023). COM: Contrastive masked-attention model for incomplete multimodal learning. Neural Netw..

[30] Lin Z., Hu B. (2023). MissModal: Increasing robustness to missing modality in multimodal sentiment analysis. Trans. Assoc. Comput. Linguist..

[31] Liu X., Zhou Y., Chu Y., Sun J., Meng Y. (2024). Modality translation-based multimodal sentiment analysis under uncertain missing modalities. Inf. Fusion.

[32] Yu Y., Wang Y., Hussein M., Hung Y. (2024). Robust multimodal federated learning for incomplete modalities. Comput. Commun..

[33] Lian Z., Chen L., Sun L., Liu B., Tao J. (2023). GCNet: Graph completion network for incomplete multimodal learning in conversation. IEEE Trans. Pattern Anal. Mach. Intell..

[34] González-Baldizón Y., Fragoso-Mandujano J.-A., Urbina-Brito N., Chandomí-Castellanos E., Bermúdez-Rodríguez J.-I., Pérez-Pérez E.-J., Guzmán-Rabasa J.-A. (2026). A hybrid algorithm for fault diagnosis in nonlinear UAV systems using conditional LSTM autoencoders. Algorithms.

